# Falls among community-residing stroke survivors following inpatient rehabilitation: a descriptive analysis of longitudinal data

**DOI:** 10.1186/1471-2318-9-46

**Published:** 2009-10-14

**Authors:** Laura M Wagner, Victoria L Phillips, Amanda E Hunsaker, Pamela G Forducey

**Affiliations:** 1 The Kunin-Lunenfeld Applied Research Unit Baycrest 3560 Bathurst Street, 736 7th Floor Toronto, ON, M6A 2E1, Canada; 2 University of Toronto, Bloomberg Faculty of Nursing, Toronto, ON, Canada; 3 Department of Health Policy and Management, Rollins School of Public Health of Emory University, Atlanta, GA, USA; 4 School of Social Work, University of Pittsburgh, Pittsburgh, PA, USA; 5 INTEGRIS Southwest Medical Center Neuroscience and TeleHealth, INTEGRIS Southwest Medical Center, Oklahoma City, OK, USA

## Abstract

**Background:**

Stroke victims are at relatively high risk for injurious falls. The purpose of this study was to document longitudinal fall patterns following inpatient rehabilitation for first-time stroke survivors.

**Methods:**

Participants (n = 231) were recruited at the end of their rehab stay and interviewed monthly via telephone for 1 to 32 months regarding fall incidents. Analyses were conducted on: total reports of falls by month over time for first-time and repeat fallers, the incidence of falling in any given month; and factors differing between fallers and non fallers.

**Results:**

The largest percentage of participants (14%) reported falling in the first month post-discharge. After month five, less than 10% of the sample reported falling, bar months 15 (10.4%) and 23 (13.2%). From months one to nine, the percentage of those reporting one fall with and without a prior fall were similar. After month nine, the number of individuals who reported a single fall with a fall history was twice as high compared to those without a prior fall who reported falling. In both cases the percentages were small. A very small subset of the population emerged who fell multiple times each month, most of whom had a prior fall history. At least a third of the sample reported a loss of balance each month. Few factors differed significantly between fallers and non-fallers in months one to six.

**Conclusion:**

Longitudinal data suggest that falls most likely linked to first time strokes occur in the first six months post discharge, particularly month one. Data routinely available at discharge does not distinguish fallers from non-fallers. Once a fall incident has occurred however, preventive intervention is warranted.

## Background

Stroke survivors are at high risk for developing a wide range of complications [1]. Common secondary conditions following a stroke include pain, depression, muscle spasms, incontinence, fatigue, second stroke, urinary tract infections, pressure ulcers, immobility, and falls [1-6]. These conditions can develop during the acute or post-acute stroke recovery period.

Falls are the most frequently reported accident among older adults [7] and some data indicate that stroke victims are especially at high risk for falls [8,9]. Results from the Behavioral Risk Factor Surveillance Survey, conducted by the Centers for Disease Control and Prevention [10] indicate that among those surveyed, 15.9% of adults 65 and older reported falling in the prior three months [11]. No information on the severity of the falls or loss of balance was available. Most studies on falls in stroke patients have focused on those falling in either the acute care or rehabilitation setting [12-18]. Few studies document the frequency of falls following discharge from the inpatient setting.

The literature on falls in community-dwelling stroke survivors is generally cross-sectional. In identified stroke populations, studies ask about falls over some time interval. In these studies the time intervals differ from the stroke event often differ and other comorbidities affecting the population are seldom identified. The longer the interval from stroke to survey the less likely it is that the fall can be definitely attributed to the stroke. Recall issues also become important. Available studies on stroke survivors in community-based populations have examined: falls only in women [19], long-term stroke survivors (greater than one year since stroke) [8,20,21], small samples of stroke survivors [9,20], or fall data with follow-up limited to six months [22-25]. We were unable to identify a study that prospectively followed fall patterns in a cohort among newly diagnosed stroke survivors for more than six months and followed on a month-to-month basis.

Understanding fall patterns and predictors of falls is important, particularly as the stroke recovery trajectory is long and unpredictable. Falls can increase a stroke victim's risk of morbidity and mortality. The risk of fall-related fractures is especially of concern in stroke survivors given their impaired mobility and high risk for developing osteoporosis [26,27]. Longitudinal data can provide important information on initial fall patterns and risk factors for falls in stroke survivors to supplement the cross-sectional literature. Potentially these data can help inform interventions to prevent stroke post discharge.

This study had two objectives: 1) to document the frequency and monthly pattern of reported falls in community residing stroke survivors over a period of up to 32 months and 2) to identify differential characteristics among fallers and non fallers.

## Methods

Data were obtained from a multi-center prospective cohort study documenting the patterns of secondary conditions, including falls, following a first-time stroke. Recruitment began in October 2000 and ended in June 2003. The study was approved by the Institutional Review Board (IRB) of Emory University and by each hospital's IRB.

### Subjects and Sites

Participants were recruited from urban, not-for-profit inpatient acute care (with a specific stroke unit) and rehabilitation hospitals in the southeastern and southwestern United States. The sites were located in low-income to upper-middle class neighborhoods and were either privately owned or university affiliated. Inclusion criteria were general to recruit as large a sample as possible. Eligible were all first-time stroke patients age 18 years or older, who were diagnosed with either hemorrhagic or ischemic stroke, had a telephone, had a caregiver who could act as a proxy respondent if speech impaired, and who were likely to be discharged to the community. The majority of subjects (94.3%) were living at home following the stroke, while the remainder had been discharged to either an assisted living or nursing home facility (n = 14). Eleven percent of those discharged to the community came from a hospital with a dedicated stroke unit.

A hospital case manager referred patients that were appropriate for the study to the project site coordinator. A site coordinator then visited the patient during their acute or rehabilitation stay to describe the project and obtain consent. All subjects voluntarily signed an approved consent form. A separate consent was available for patient family members if subjects were unable to sign or deemed unable to understand the requirements of the study. Researchers believed that caregiver reports of falls and fall-related health care utilization would be reliable for those who could not self-report. Subjects were called the first week post-discharge to review the study and contacted monthly thereafter via telephone.

### Measures

Data were collected from two sources. We gathered information from discharge inpatient hospital medical records as well as from monthly interviews conducted with the participants. Our goal in this approach was to identify whether information routinely available at discharge could predict future outcomes (e.g., falls) following discharge.

### Medical Record Review

Following consent, baseline data on demographics, past medical history, and discharge Functional Independence Measure (FIM) scores were collected from inpatient medical records. The FIM is the most widely accepted instrument to assess progress during inpatient rehabilitation and has been used to predict stroke rehabilitation outcomes [5,28]. This instrument assesses a patient's level of independence in bathing, grooming, bowel and bladder control, transfers, ambulation, and communication. The FIM ranges from 18-126 with a higher score reflecting greater independence and includes motor and cognitive subscales [29]. The reliability and validity of the FIM have been studied extensively [30-32].

### Monthly Interviews

Subjects were followed monthly post discharge and completed detailed telephone interviews regarding secondary conditions experienced in the previous month. All interviewers were trained at the Emory University Rollins School of Public Health by the study's principal investigator (VLP) and project coordinator (AEH). Given rolling recruitment, subjects entered the study as they were affected by strokes and discharged over time. They participated in the study for intervals varying from six to thirty-two months. If a subject completed two years and agreed to continue, their interview schedule changed to quarterly interviews.

Subjects were mailed a notebook to be used for the study. The notebook included laminated sheets of the questions that would be covered and calendar pages that noted the weeks when they would be called. Subjects were also encouraged to use the calendar to mark days when a fall occurred. If the subject was not available in a particular month, then they were asked through a prompting process during the interview to recall the previous month and to refer to their calendar.

Falls ("a fall report") were defined as a fall to the ground and were only self-reported. Subjects were first asked to report whether they had a fall in the past month. If the subject reported a fall they were then asked the date of occurrence. Subjects were then asked whether they had experienced any more falls and questioned about each subsequent fall. Episodes of near-falls [33] were not identified per se, however, subjects were asked whether they had a loss of balance in the past four weeks as a prelude to a "full" fall report. Loss of balance [34] is defined as a loss in the center of gravity, without a fall to the ground.

### Statistical Analysis

All statistical analyses were undertaken with SPSS package for Windows, version 11.5 (SPSS, Inc., Chicago, IL). Statistical significance was defined at p < 0.05 unless otherwise specified. Standard descriptive statistics were used to describe the sample, while Chi-square tests for categorical variables; t-tests (non-skewed) and Mann-Whitney U tests (skewed) for continuous variables were used to compare significant differences between those who did and did not fall. Hazard rate regression was used to identify the likelihood of a respondent falling in a given month, based on their individual reporting times [35].

## Results

### Sample Characteristics

A total of 290 subjects were referred for recruitment and 245 subjects consented and completed at least one interview. Ultimately, 14 subjects were discharged to nursing homes and were not included in the analyses. Those discharged to a long term care setting had significantly lower FIM scores (74.54 vs. 97.63) (t = 4.76; df = 224; p = .001) and were significantly older (64.13 vs. 72.78 years) (t = 2.19; df = 243; p = 0.03) compared to those who were discharged home after their rehabilitation stay. Thus, our results include data from 231 participants. The mean follow-up period was 13.50 months (median 13; range 1-32). Thirty-nine subjects (16.8% of the sample) dropped out of the study during the two-year follow-up period. Reasons for drop out included loss to follow-up (48.7%), death (28.2%), or they no longer wanted to participate (15.4%).

Table 1 includes demographics information of the sample and other data points routinely available at discharge. Overall, subjects ranged in age from 21-92 years (mean ± SD age, 64.13 ± 14.59 years). Most subjects (72.7%) were Caucasian and just over half (55.1%) were females. Over half (53.1%) had post-secondary school education. Married subjects accounted for 60.9% of the sample. Almost half (45.9%) of the subjects were retired prior to the stroke event.

**Table 1:** Demographic and Discharge Comorbidity Data

	**Range**	**Mean ± SD**
Age	21 - 92 years	64.13+14.59 years
Length of Stay	0 - 89 days	20.26+15.93 days
Total FIM Score at Discharge	27 - 126	97.63+16.99
Motor FIM Subscale	19 - 91	67.92+14.10
Cognitive FIM Subscale	8 - 35	29.28+5.52
Race: White - n (%)	168 (72.7)	
Sex: female - n (%)	127 (55.0)	
Marital Status - n (%)		
Married	143 (61.9)	
Widowed	46 (19.9)	
Other (single, divorced, separated, or have significant other)	42 (18.2)	
Education - n (%)		
High School or less	91 (39.4)	
College or Tech School	98 (42.2)	
Graduate School	5 (2.2)	
Employment - n (%)		
Retired	105 (45.9)	
Full-time	86 (37.6)	
Other (part-time, looking for work, homemaker, student, or unemployed)	38 (16.5)	
Comorbidities - n (%)		
Hypertension	126 (54.5)	
Diabetes	49 (21.2)	
Communication Disorder	51 (22.1)	
Lipid Disorder	46 (19.9)	
Hemiparesis/Hemiplegia	38 (16.5)	
Type of Stroke - n (%)		
Ischemic	197 (85.3)	
Hemorrhagic	34 (14.7)	
Side of Stroke		
Left Hemisphere	90 (39.0)	
Right Hemisphere	90 (39.0)	
Other (e.g., undefined, cerebellar, brainstem)	51 (22.1)	

1 FIM: Functional Independence Measure

The most common discharge diagnoses following the stroke included hypertension (54.5%); diabetes (21.2%), communication disorder (22.1%); lipid disorders (19.9%); and hemiparesis or hemiplegia (16.5%). Ischemic strokes accounted for 85.3% and strokes occurred in both the right hemisphere (39%) and left hemisphere (39%).

The mean length of stay in rehabilitation was 20.25 days (median 18; range 0-89). The mean total FIM score was 97.63 (median 101; range 27-126). The group was relatively highly functional physically and moderately functional cognitively compared to other acute rehabilitation stroke survivors [28,36].

As part of the subjects' discharge plan, over two-thirds (68.8%) of the subjects were receiving some type of therapy in the first three months following discharge. Half of these subjects (n = 116) were receiving two or more therapies. The most common therapies received were outpatient physical therapy (45.5%), outpatient occupational therapy (39.4%), and outpatient speech therapy (21.6%).

### Fall Reports

The sample reported 335 total fall incidents (259.83 person-years observation) [37]. These data include reports of single and multiple falls during each interview period. Table 2 provides the overall fall results for the sample, while Table 3 breaks down the data into types of fall patterns. Based on hazard rate analysis, which adjusts for the observation period for each individual respondent, a stroke survivor's chance of falling in any given month is 11%.

**Table 2:** Reported Falls and Loss of Balance by Month for all Participants

**Month After** **Discharge**	**Number of** **Respondents**	**Number of Reported** **Fall Incidents**^**1**^	**Percent of Sample** **Reporting no Falls** **n (%)**	**Loss of Balance** **n (%)**
1	189	50	155 (82.0)	105 (55.6)
2	194	30	173 (89.2)	90 (46.4)
3	190	31	165 (86.8)	93 (48.9)
4	189	28	164 (86.8)	83 (43.9)
5	179	22	157 (87.7)	78 (43.6)
6	166	10	159 (95.2)	60 (35.9)
7	160	15	147 (91.9)	71 (44.4)
8	151	7	144 (95.4)	63 (41.4)
9	145	5	140 (96.6)	48 (33.1)
10	140	19	128 (91.4)	53 (37.9)
11	125	12	116 (92.8)	52 (41.6)
12	125	16	113 (90.4)	55 (44.0)
13	116	4	112 (96.6)	48 (41.4)
14	109	15	99 (90.8)	51 (46.8)
15	96	17	83 (86.5)	43 (44.8)
16	83	6	78 (94.0)	40 (48.2)
17	76	11	67 (88.2)	34 (44.7)
18	70	12	63 (90.0)	37 (52.9)
19	58	2	56 (96.6)	25 (43.1)
20	52	2	50 (96.2)	20 (38.5)
21	44	1	43 (97.7)	18 (40.9)
22	43	3	40 (93.0)	23 (53.5)
23	38	5	33 (86.8)	20 (52.6)
24	32	3	29 (90.6)	13 (40.6)
27	27	7	23 (85.2)	11 (40.7)
30	21	1	20 (95.2)	9 (42.9)

^1 ^Fall incident is defined as fall to the ground.

**Table 3:** Participants Reports of Single and Multiple Fall Reports by Month

**Month After Discharge**	**Participants Reporting a Single Fall with No Fall history** **n (%)**	**Participants Reporting a Single Fall with a Prior Fall(s)** **n (%)**	**Participants Reporting Multiple Falls with no Prior Falls** **n (%)**	**Participants Reporting Multiple Falls with a Prior, Fall(s)** **n (%)**
1	26 (13.8)	-	8 (4.2)	-
2	11 (5.7)	4 (2.1)	5 (2.6)	1 (0.5)
3	13 (6.8)	8 (4.2)	1 (0.5)	3 (1.6)
4	10 (5.3)	12 (6.3)	-	3 (1.6)
5	6 (3.4)	15 (8.4)	-	1 (0.6)
6	3 (1.8)	4 (2.4)	-	1 (0.6)
7	6 (3.8)	6 (3.8)	-	1 (0.6)
8	3 (2.0)	4 (2.6)	-	-
9	3 (2.1)	2 (1.4)	-	-
10	3 (2.1)	7 (5.0)	-	2 (1.4)
11	1 (0.8)	6 (4.8)	-	2 (1.6)
12	2 (1.6)	6 (4.8)	1 (0.8)	3 (2.4)
13	3 (2.6)	1 (0.9)	-	-
14	3 (2.8)	4 (3.7)	-	3 (2.8)
15	2 (2.1)	8 (8.3)	-	3 (3.1)
16	-	4 (4.8)	-	1 (1.2)
17	1 (1.3)	6 (7.9)	-	2 (2.6)
18	2 (2.9)	4 (5.7)	-	1 (1.4)
19	-	2 (3.4)	-	-
20	-	2 (3.8)	-	-
21	-	1 (2.3)	-	-
22	-	3 (7.0)	-	-
23	1 (2.6)	4 (10.5)	-	-
24	-	3 (9.4)	-	-
27	1 (3.7)	1 (3.7)	-	2 (7.4)
30	-	1 (4.8)	-	-

Note, again due to rolling enrollment, the larger number of respondents with data in early months (i.e. one to six) reflects the fact that subjects were recruited over time and those recruited late in the study were followed for a shorter period of time. Participants who missed an interview month were prompted to during the call to report fall incidents in the prior month through anchoring questions, such as a holiday, birthday or a health care visit reported in the prior month.

Table 2 shows the number of fall incidents for the sample population. The largest percentage of the sample reported falls during the first five months post discharge. The largest number of fall incidents (n = 50) occurred in month one. These fall incidents involved 16% of the respondents; 82% reported no falls. After month five post-discharge, less than 10% of the sample reported falling in any month, bar months 15 (13.5%), 17 (11.8%), and 23 (13.2%).

Table 3 disaggregates fall episodes into those reporting single and multiple falls and whether or not the participant had fallen in a prior month. From month three on, those reporting a single fall with a prior fall generally exceeded those reporting a single fall with no fall history. From months three to eight, those reporting one fall per month with and without a prior fall were similar. After month nine, twice as many participants reporting a single fall had a fall history (bar month 9) compared to those without a prior fall.

Two very small groups emerged in relation to reports of multiple falls. The percentage of the population reporting multiple falls was extremely small. Three of the four reports of multiple falls (in those without a prior history) occurred in the first three months following discharge. Also, a very small subset of the population emerged who fell multiple times each month; most of whom had a prior fall history.

Table 4 compares characteristics of fallers versus non-fallers in months one and six as this was the time period when most falls occurred. Factors compared show the data available routinely at discharge. Very few factors differed significantly between fallers and non-fallers in months one to six. Length of rehab stay was significantly longer in fallers and those experiencing a loss of balance in the first month following discharge. In month 6, there was a significant relationship between those with a communication disorder and falling. Additionally, type of stroke (e.g. ischemic) emerged having a significant relationship in those who had ever fallen by month 6 during the follow-up period.

**Table 4:** Characteristics of Fallers and non-Fallers by Month

	**Fall (Month 1)**		**Loss of Balance (Month 1)**		**Fall (Month 6)**		**Ever Fallen (Month 6)**		**Loss of Balance (Month 6)**	
	**Yes**	**No**	**Yes**	**No**	**Yes**	**No**	**Yes**	**No**	**Yes**	**No**
FIM total	98.13	99.86	97.75	101.84	94.62	100.32	99.45	100.34	94.80	102.97
mean (SD)^$^	(14.74)	(17.70)	(16.12)	(18.30)	(28.26)	(16.00)	(16.36)	(17.09)	(20.17)	(13.62)
FIM motor	67.87	69.89	68.20	71.28	65.75	70.35	69.20	70.62	66.83	71.92
mean (SD)^$^	(12.22)	(14.43)	(12.57)	(15.70)	(20.40)	(13.25)	(13.08)	(14.08)	(15.38)	(12.24)
FIM cognitive	29.55	29.48	29.01	30.14	28.88	29.56	29.14	30.15	28.43	30.17
mean (SD) $	(5.33)	(5.87)	(6.10)	(5.23)	(9.01)	(5.37)	(5.57)	(5.59)	(6.39)	(4.94)
Length of rehab stay in days	18.21	23.85*	21.69*	16.14	19.13	18.49	16.57	21.98*	23.42	15.77
mean (SD) ‡	(15.69)	(15.35)	(15.15)	(16.01)	(13.57)	(15.23)	(14.46)	(15.75)	(17.57)	(12.76)
Discharge comorbidities										
Hypertension (%)^†^	16 (16.3)	82 (83.7)	52 (53.1)	46 (46.9)	2 (2.5)	77 (97.5)	27 (34.2)	52 (65.8)	26 (32.5)	54 (67.5)
Diabetes (%)^†^	9 (25.0)	27 (75.0)	14 (38.9)	22 (61.1)	1 (3.4)	28 (96.6)	13 (44.8)	16 (55.2)	13 (44.8)	16 (55.2)
Communication disorder (%)^†^	6 (16.7)	30 (83.3)	19 (52.8)	17 (47.2)	4 (12.9)*	27 (87.1)*	9 (29.0)	22 (71.0)	12 (37.5)	20 (62.5)
Lipid disorder (%)^†^	5 (13.5)	32 (86.5)	20 (54.1)	17 (45.9)	1 (3.1)	31 (96.9)	10 (31.2)	22 (68.8)	10 (31.2)	22 (68.8)
Hemiparesis/plegic (%)^†^	5 (25.0)	15 (75.0)	10 (50.0)	10 (50.0)	2 (10.0)	18 (90.0)	8 (40.0)	12 (60.0)	9 (45.0)	11 (55.0)
Type of stroke										
Ischemic (%)	27 (16.8)	134 (83.2)	88 (54.7)	73 (45.3)	6 (4.2)	136 (95.8)	47 (33.1)	95* (66.9)	51 (35.7)	92 (64.3)
Hemorrhagic (%)^†^	7 (25.0)	21 (75.0)	17 (60.7)	11 (39.3)	2 (8.3)	22 (91.7)	13 (54.2)	11* (45.8)	9 (37.5)	15 (62.5)
CVA Location										
Right (%)^†^	15 (20.3)	59 (79.7)	37 (50.0)	37 (50.0)	4 (6.0)	63 (94.0)	29 (43.3)	38 (56.7)	26 (38.2)	42 (61.8)
Left (%)^†^	14 (19.4)	58 (80.6)	46 (63.9)**	26 (36.1)	1 (1.6)	63 (98.4)	20 (31.2)	44 (68.8)	19 (29.7)	45 (70.3)
Other/Undifferentiated (%)^†^	5 (11.6)	38 (88.4)	22 (51.2)	21 (48.8)	3 (8.6)	32 (91.4)	11 (31.4)	24 (68.6)	15 (42.9)	20 (57.1)

FIM = Functional Independence Measure

^$^Mann-Whitney U test

^†^Chi Square of Independence test

^‡^Student's t-test

*significance at p = 0 < .05

**significance = 0.05 < p < 0.10

## Discussion

Preventing falls in people affected by stroke is an important healthcare goal [38]. Understanding when falls occur following discharge into the community is key to prevention and to timing appropriate prevention interventions to maximize their effectiveness. To our knowledge, this is the first study to longitudinally follow fall occurrences monthly among newly discharged first-time stroke survivors, past six months following discharge from rehabilitation. Longitudinal data provide additional information to supplement cross-sectional data in understanding the relationship between stroke events and falls. The analyses presented here examined fall patterns descriptively and in detail for stroke patients prospectively for up to two and a half years following discharge.

At least a third of the sample reported a loss of balance over the observation period, while a much smaller percentage reported a fall to the ground - in month one 18% reported a fall, 56% a loss of balance. These findings are in concert with recent data on people age 65 and older reported by the CDC, although the comorbidities of the population surveyed were not described. Here, the majority of falls are concentrated in the first six months post discharge and after that period those with a single falls begin to develop a fall history, with multiple falls being less common than single falls. Of note is that the percentage of the sample reporting falls, multiple or single in a given period, was extremely small - less than 10% in four out of 15 months of observation from month 6 on.

Data here suggest that information routinely available at discharge does little to assist in helping to identify first-time fallers. This finding is consistent with the work of Ray et al. [39]who through a multi-faceted intervention in nursing homes significantly affected the likelihood of a second fall occurring, but did not significantly affect the likelihood of a first time fall.

Based on demographics, our sample is generalizable to the community-dwelling stroke survivor population. The sample is similar to national samples in gender and racial mix [40,41], while average age was lower than for national populations [41]. The ratio of ischemic to hemorrhoragic strokes was also similar to national surveys [42,32]. Studies that report on medical rehabilitation facilities subscribing to the Uniform Data System for Medical Rehabilitation reported age at stroke onset ranging from 70-71 years [29,44,45], while the mean age for our sample was 65 years. Studies also report lower discharge total FIM scores, ranging from 86.5-87.2 [43-45]. The mean discharge FIM for this study was 96.3. Furthermore, our sample had a lower (16.5%) percentage of hemiparesis compared to other large studies (50%) [46]. This could likely be attributable to the lower nursing home placement, higher functional status, and lower age characteristics of our group. The effect of a lower age is unclear. It is possible that fewer falls occurred as respondents could have less age-related morbidity at the time of the stroke.

This study documents that falls, clearly linked to strokes, occur in the first several months following discharge from acute rehabilitation. The frequency of falls in this sample in the first six months is lower than the percentages in many cross-sectional studies in which other comordities may have influenced the fall rate [22-25]. The difference is likely attributable to the fact that our analysis focuses on the timing of and factors related to stroke for survivors during a specific window since injury, compared to other studies which look at stroke survivors whose time since stroke varies greatly or when follow-ups occur less than monthly, increasing the likelihood of recall bias. Additionally, as mentioned above, our sample was somewhat younger in mean age, resulting in a decreased risk. While the majority of falls occurred in the first five months post-discharge, falls continued to occur monthly for a small subset of the population throughout the 2.5 year follow-up period. However, it is important to note that the further the fall incident is from the time of the stroke, the less likely that it can be attributed with certainty to the index stroke. Few comorbid characteristics of the data available at discharge identified differences between fallers and non-fallers. Differences were noted namely length of stay, communication disorder, and type of stroke (e.g., ischemic). Factors such as altered gait patterns in patients while performing cognitive linguistic tasks such as walking all has the potential to increase fall risk [47] in those with communication disorders. Increased length of stay was a significant factor in falling likely because those who were more severely affected by the stroke (and thus prone to falls) required longer inpatient treatment. Logistic modeling with these potential risk factors failed to identify any meaningful predictors for falling.

These data suggest that predicting falls post-discharge based solely on the medical record data routinely available (e.g., age, functional status) [48] at discharge is extremely difficult. Other scores, difficult for clinicians to observe, such as housing arrangements and levels of support post discharge with activities of daily living (ADL) support, may play a role in affecting falls, particularly first-time falls. Also, the post-discharge therapy recommendations may also play a role. Expanding the type of data at discharge for clinicians to consider may help reduce the occurrence of falls in the early period following discharge.

There were a few additional limitations to this study. Fall incidents were self-reported and while this methodology in commonly used in cross-sectional studies, the effect of this reporting mechanism on reports is unknown. Furthermore, the definition here of "fall to the ground" leads to a conservative estimate of fall incidents. Other definitions, such as likely falls or falls prevented by participant response, such as a nearby object or a caregiver intervening, may lead to different results. We also had no way to assess the severity of the fall. We also did not collect data on participants' "fear of falling"[49], nor on the circumstances surrounding the fall [20,24], potentially important areas for further inquiry.

## Conclusion

In conclusion, falls in community-dwelling stroke survivors are a frequent occurrence following in patient rehabilitation. Further analysis of the key characteristics that increased length of rehabilitation stay as well as the occurrence of near-fall events such as loss of balance may increase our understanding of predictive factors for falls among stroke survivors following discharge to the community. Longitudinal data are useful to supplement cross-sectional data available on stroke populations in the continuing effort to design effective interventions to prevent falls among people affected by strokes.

## Competing interests

The authors declare that they have no competing interests.

## Authors' contributions

LMW was the primary author. She conducted data analysis, and oversaw preparation of the manuscript. VLP was the Principal Investigator. She also drafted parts of the manuscript, mentored the primary author in the research process, and edited the manuscript. AEH: implemented the study, conducted data analysis, and assisted with preparation of the manuscript. PGF contributed to the initial study design, recruited subjects, collected data and reviewed drafts of the manuscript. All authors read and approved the final manuscript.

## Pre-publication history

The pre-publication history for this paper can be accessed here:


